# Supplementary material for: The relationship between physical activity and sleep quality among college students: The chain-mediating effects of self-control and mobile phone addiction
Source: PLoS One. 2024 Dec 19;19(12):e0315930. doi: 10.1371/journal.pone.0315930 (PMC11658481; doi:10.1371/journal.pone.0315930)
Supplement: Attachment — Submitted filename: PONE-D-24-34938.docx [file pone.0315930.s002.docx]

**PLOS ONE**

**The relationship between physical activity and sleep quality among college students: The chain-mediating effects of self-control and mobile phone addiction**

--Manuscript Draft--

| **Manuscript Number:** | PONE-D-24-34938 |
| --- | --- |
| **Article Type:** | Research Article |
| **Full Title:** | The relationship between physical activity and sleep quality among college students: The chain-mediating effects of self-control and mobile phone addiction |
| **Short Title:** | The relationship between physical activity and sleep quality among college students |
| **Corresponding Author:** | Chengbo Yang  Chengdu Sport University chengdu, Sichuan CHINA |
| **Keywords:** | physical activity；self-control；mobile phone addiction；sleep quality |
| **Abstract:** | The psychological and physiological health of undergraduates was correlated with the sleep quality, which can be improved through increasing physical activity. However, the correlations between physical activity and sleep quality are subject to various factors. In this study, we investigated the effects of self-control and mobile phone addiction on the correlations between physical activity on undergraduates’ sleep quality at the psychological and behavioral levels. Data was collected through a survey with a convenient sample of 2,274 students in China. The study utilized scales of physical activity, sleep quality, self-control, and mobile phone addiction to quantitatively evaluate the impact of physical activity on the sleep quality of undergraduates. The correlations were analyzed using SPSS 26.0, including descriptive statistics, confidence tests, common method bias tests, correlation analysis, and hypothesis tests. Pearson correlation analysis shows that physical activity was significantly correlated with sleep quality (r= -0.541, p < 0.001), and that physical activity and sleep quality were significantly correlated with self-control and mobile phone addiction.  Regression analysis shows that physical activity had a significant positive regression effect on self-control (standardized regression coefficient β = 0.234, p < 0.001), a significant negative regression effect on mobile phone addiction (β = –0.286, p < 0.001), and a significant negative regression effect on sleep quality (β = –0.351, p < 0.001). Further, a chain mediation model of physical activity → self-control → mobile phone addiction → sleep quality was proposed. The findings provide basic data for college students to promote physical activity and improve sleep quality. |
| **Order of Authors:** | Zhonggen Yin, PHD |
|  | Chengbo Yang, PHD |
|  | Tong Liu, PHD |
|  | Jialiang Yu, PHD |
|  | Xiaomei Yu |
|  | Shuting Huang |
|  | Yanhong Zhang |
| **Additional Information:** |  |
| **Question** | **Response** |
| **Financial Disclosure**  Enter a financial disclosure statement that describes the sources of funding for the work included in this submission. Review the [submission guidelines](http://journals.plos.org/plosone/s/submission-guidelines#loc-financial-disclosure-statement) for detailed requirements. View published research | Yes |

| articles from [PLOS ONE](https://journals.plos.org/plosone/) for specific examples.  This statement is required for submission and **will appear in the published article** if the submission is accepted. Please make sure it is accurate. |  |
| --- | --- |
| **Funded studies**  Enter a statement with the following details:   - Initials of the authors who received each award - Grant numbers awarded to each author - The full name of each funder - URL of each funder website - Did the sponsors or funders play any role in the study design, data collection and analysis, decision to publish, or preparation of the manuscript? |  |
| Did you receive funding for this work? |  |
| Please add funding details.  as follow-up to "**Financial Disclosure**  Enter a financial disclosure statement that describes the sources of funding for the work included in this submission. Review the [submission guidelines](http://journals.plos.org/plosone/s/submission-guidelines#loc-financial-disclosure-statement) for detailed requirements. View published research articles from [PLOS ONE](https://journals.plos.org/plosone/) for specific examples.  This statement is required for submission and **will appear in the published article** if the submission is accepted. Please make sure it is accurate. | Yanhong Zhang received the award. The full name of funder: The Scientific and Technological Research Program of Chongqing Municipal Education Commission (Grant No. KJZD-K202200306).URL of funder website:https://jw.cq.gov.cn/.Zhang Yanhong participated in the data collection and analysis of the paper. |

| **Funded studies**  Enter a statement with the following details:   - Initials of the authors who received each award - Grant numbers awarded to each author - The full name of each funder - URL of each funder website - Did the sponsors or funders play any role in the study design, data collection and analysis, decision to publish, or preparation of the manuscript? |  |
| --- | --- |
| Did you receive funding for this work?" |  |
| Please select the country of your main research funder (please select carefully as in some cases this is used in fee calculation).  as follow-up to "**Financial Disclosure**  Enter a financial disclosure statement that describes the sources of funding for the work included in this submission. Review the [submission guidelines](http://journals.plos.org/plosone/s/submission-guidelines#loc-financial-disclosure-statement) for detailed requirements. View published research articles from [PLOS ONE](https://journals.plos.org/plosone/) for specific examples.  This statement is required for submission and **will appear in the published article** if the submission is accepted. Please make sure it is accurate. | CHINA - CN |
| **Funded studies**  Enter a statement with the following details:   - Initials of the authors who received each award - Grant numbers awarded to each author - The full name of each funder - URL of each funder website - Did the sponsors or funders play any role in the study design, data collection and analysis, decision to publish, or preparation of the manuscript? |  |
| Did you receive funding for this work?" |  |
| **Competing Interests** | The authors have declared that no competing interests exist. |

| Use the instructions below to enter a competing interest statement for this submission. On behalf of all authors, disclose any [competing interests](http://journals.plos.org/plosone/s/competing-interests) that could be perceived to bias this work—acknowledging all financial support and any other relevant financial or non- financial competing interests.  This statement is required for submission and **will appear in the published article** if the submission is accepted. Please make sure it is accurate and that any funding sources listed in your Funding Information later in the submission form are also declared in your Financial Disclosure statement.  View published research articles from  [PLOS ONE](http://journals.plos.org/plosone/) for specific examples. |  |
| --- | --- |
| **NO authors have competing interests**  Enter: The authors have declared that no competing interests exist.  **Authors with competing interests**  Enter competing interest details beginning with this statement:  I have read the journal's policy and the authors of this manuscript have the following competing interests: [insert competing interests here] |  |
| * typeset |  |
| **Ethics Statement**  Enter an ethics statement for this submission. This statement is required if the study involved:   - Human participants - Human specimens or tissue - Vertebrate animals or cephalopods - Vertebrate embryos or tissues | The studies involving human participants were reviewed and approved by the Ethics Committee of Chengdu Sport University (2023-150). The participants were informed that partaking in the study was voluntary and that completing the questionnaire via an online platform indicated their informed consent to voluntarily participate in the study. The participants were to read the research descriptions and consent form and agree to participate, and the survey was designed to end automatically if they did not agree.  Once the questionnaire has been submitted, the author has permanent rights to the data. The questionnaire included the purpose and method of research, anonymity and confidentiality regarding participation, and the choice to agree or withdraw from participation, as well as the confirmation that the survey will not be used for purposes |

| - Field research   Write "N/A" if the submission does not require an ethics statement.  General guidance is provided below. Consult the [submission guidelines](http://journals.plos.org/plosone/s/submission-guidelines#loc-guidelines-for-specific-study-types) for detailed instructions. **Make sure that all information entered here is included in the Methods section of the manuscript.** | other than research. |
| --- | --- |

| **Format for specific study types**  **Human Subject Research (involving human participants and/or tissue)**   - Give the name of the institutional review board or ethics committee that approved the study - Include the approval number and/or a statement indicating approval of this research - Indicate the form of consent obtained (written/oral) or the reason that consent was not obtained (e.g. the data were analyzed anonymously)   **Animal Research (involving vertebrate animals, embryos or tissues)**   - Provide the name of the Institutional Animal   Care and Use Committee (IACUC) or other relevant ethics board that reviewed the study protocol, and indicate whether they approved this research or granted a formal waiver of ethical approval   - Include an approval number if one was obtained - If the study involved non-human primates, add additional details about animal welfare and steps taken to ameliorate suffering - If anesthesia, euthanasia, or any kind of animal sacrifice is part of the study, include briefly which substances and/or methods were applied   **Field Research**  Include the following details if this study involves the collection of plant, animal, or other materials from a natural setting:   - Field permit number - Name of the institution or relevant body that granted permission |  |
| --- | --- |
|  |  |
| **Data Availability**  Authors are required to make all data underlying the findings described fully available, without restriction, and from the time of publication. PLOS allows rare exceptions to address legal and ethical concerns. See the [PLOS Data Policy](http://journals.plos.org/plosone/s/data-availability) and [FAQ](http://journals.plos.org/plosone/s/data-availability#loc-faqs-for-data-policy) for detailed information. | Yes - all data are fully available without restriction |

| A Data Availability Statement describing where the data can be found is required at submission. Your answers to this question constitute the Data Availability Statement and **will be published in the article**, if accepted. |  |
| --- | --- |
| **Important:** Stating ‘data available on request from the author’ is not sufficient. If your data are only available upon request, select ‘No’ for the first question and explain your exceptional situation in the text box. |  |
| Do the authors confirm that all data underlying the findings described in their manuscript are fully available without restriction? |  |
| **Describe where the data may be found in full sentences. If you are copying our sample text, replace any instances of XXX with the appropriate details.**   - If the data are **held or will be held in a public repository**, include URLs, accession numbers or DOIs. If this information will only be available after acceptance, indicate this by ticking the box below. For example: All XXX files are available from the XXX database (accession number(s) XXX, XXX.). - If the data are all contained **within the**   **manuscript and/or Supporting Information files**, enter the following: All relevant data are within the manuscript and its Supporting Information files.   - If neither of these applies but you are able to provide **details of access elsewhere**, with or without limitations, please do so. For example:   Data cannot be shared publicly because of [XXX]. Data are available from the XXX Institutional Data Access / Ethics Committee (contact via XXX) for researchers who meet the criteria for access to confidential data.  The data underlying the results presented in the study are available from (include the name of the third party | All relevant data are within the manuscript and its Supporting Information files. |

| and contact information or URL).   - This text is appropriate if the data are owned by a third party and authors do not have permission to share the data.   * typeset |  |
| --- | --- |
| Additional data availability information: | Tick here if the URLs/accession numbers/DOIs will be available only after acceptance of the manuscript for publication so that we can ensure their inclusion before publication.; Tick here if your circumstances are not covered by the questions above and you need the journal’s help to make your data available. |

Manuscript	[Click here to access/download;Manuscript;Manuscript.docx](https://www2.cloud.editorialmanager.com/pone/download.aspx?id=37280948&guid=c23194c2-f6ed-4646-9511-9c34a3686b34&scheme=1)[<https://www2.cloud.editorialmanager.com/pone/download.aspx?id=37280948&guid=c23194c2-f6ed-4646-9511-9c34a3686b34&scheme=1>](https://www2.cloud.editorialmanager.com/pone/download.aspx?id=37280948&guid=c23194c2-f6ed-4646-9511-9c34a3686b34&scheme=1)

1 The relationship between physical activity and sleep
2 quality among college students: The chain-mediating
3 effects of self-control and mobile phone addiction

### 4 Zhonggen Yin^1,2^, Chengbo Yang^1*^,Tong Liu^1^, Jialiang Yu^1^, Xiaomei Yu^1,2^,

5 Shuting Huang^3^, Yanhong Zhang^4^
6 ^1^ Institute of Sport Training, Chengdu Sport University, Chengdu, Sichuan, China
7 ^2^College of Physical Education and Health Management, Chongqing University of Education, Nanan,
8 Chongqing, China
9 ^3^Chongqing Yucai Secondary School, Jiulongpo, Chongqing, China
10 ^4^Sports Department, Southwest University of Political Science & Law, Shapingba, Chongqing, China
11 ^*^ [329628865@qq.com](mailto:329628865@qq.com)

# 12 Abstract

13 The psychological and physiological health of undergraduates was correlated with the sleep quality,
14 which can be improved through increasing physical activity. However, the correlations between
15 physical activity and sleep quality are subject to various factors. In this study, we investigated the
16 effects of self-control and mobile phone addiction on the correlations between physical activity on
17 undergraduates’ sleep quality at the psychological and behavioral levels. Data was collected through
18 a survey with a convenient sample of 2,274 students in China. The study utilized scales of physical
19 activity, sleep quality, self-control, and mobile phone addiction to quantitatively evaluate the impact
20 of physical activity on the sleep quality of undergraduates. The correlations were analyzed using
21 SPSS 26.0, including descriptive statistics, confidence tests, common method bias tests, correlation
22 analysis, and hypothesis tests. Pearson correlation analysis shows that physical activity was
23 significantly correlated with sleep quality (r= -0.541, p < 0.001), and that physical activity and sleep
24 quality were significantly correlated with self-control and mobile phone addiction. Regression
25 analysis shows that physical activity had a significant positive regression effect on self-control
26 (standardized regression coefficient β = 0.234, p < 0.001), a significant negative regression effect on
27 mobile phone addiction (β = –0.286, p < 0.001), and a significant negative regression effect on sleep
28 quality (β = –0.351, p < 0.001). Further, a chain mediation model of physical activity → self-
29 control → mobile phone addiction → sleep quality was proposed. The findings provide basic data
30 for college students to promote physical activity and improve sleep quality.

# 31 1.Introduction

32 Sleep is an important aspect of human life and is essential for maintaining one’s physical and mental
33 health[1]. However, sleep problems are prevalent throughout the world[2]. For example, in the
34 United States, 500,000 ~ 700,000 adults suffer from sleep disorders[3]. In China, the overall
35 prevalence of insomnia is 17 percent[4]. Notably, the quality of sleep in the college population has
36 been one of the major concerns of scholars [5, 6]. The sleep quality of university students is generally
37 poor, rendering them susceptible to issues such as sleep deprivation, sleep disorders and sleep
38 irregularities. These problems not only affect their studies and personal lives, but also jeopardize their
39 physical and mental health[7]. In some provinces of China, the proportion of undergraduates with
40 poor sleep quality has surpassed 20.0%; for example, the proportions in Guizhou, Hong Kong and
41 Jiangshu were 53.7%, 57.5% and 26.64%, respectively[8-10].
42 Numerous studies have shown that physical activity can have a beneficial effect on sleep quality[11,
43 12]. In a study of adolescents, a moderate amount of physical activity was observed to be associated
44 with better sleep quality[13]. A randomised controlled trial (RCT) reported that moderate-intensity
45 physical activity was associated with a significant reduction in insomnia[14]. A cross-sectional
46 research study conducted in Japan suggests that aerobic and relaxation exercise, along with reduced
47 screen time, are important for improving subjective sleep quality in university students[15]. Overall,
48 physical activity exhibited promising outcomes for improving sleep quality across diverse
49 populations.
50 Although a number of randomised controlled trials and cross-sectional survey studies have examined
51 the effects of physical activity on sleep quality, the underlying mechanisms of this process need to be
52 further explored. Additionally, most of these studies have been conducted among adults in developed
53 countries[16]. A recent meta-analysis also showed that the overall effect of physical activity on sleep
54 quality was not significant and that physical activity had no significant effect on sleep quality in
55 young people[17]. Therefore, we conducted a cross-sectional study of a group of university students
56 in China to investigate the underlying mechanisms linking physical activity and sleep quality among
57 young people in developing countries, which can help to understand the relationship between
58 physical activity and sleep quality, as well as to design interventions to improve the quality of sleep
59 and promote physical and mental health.

## 60 1.1 Correlations between physical activity and sleep quality

61 Sleep quality is an important factor in maintaining health and improving well-being[18]. Sleep
62 quality has been defined as an individual’s subjective perception of sleep [19]. Some studies have
63 shown that good sleep quality is strongly positively associated with cardiovascular health, metabolic
64 health, immune system health, and cognitive function, including reduced risk of cardiovascular
65 disease, lower incidence of metabolic syndrome[20], reduced likelihood of Alzheimer’s disease[21],
66 etc. Unfortunately, poor sleep quality has been a common phenomenon among college students,
67 although poor sleep quality can lead to lower academic performance and mental health problems in
68 students. According to a survey conducted by a mid-Atlantic university in the United States, 69
69 percent of college students are classified as poor sleepers [5]. A cross-sectional study found that only
70 48.7% of German university students reached the Pittsburgh Sleep Quality Index(PSQI) threshold for
71 poor sleep quality [22]. The prevalence of poor sleep quality among Chinese college students was
72 39.42% [23].
73 Physical activity has been demonstrated to positively impact sleep quality. A systematic review study
74 has shown that moderate physical activity benefits sleep quality in healthy people of all age groups
75 [24]. A recent systematic review study also concluded that regular physical activity can improve sleep
76 quality, reduce sleep latency, and improve overall sleep quality [25]. Higher levels of physical
77 activity were associated with better sleep quality and quantity in insomniacs [26], sedentary middle-
78 aged adults [27], and people with major depression [28]. Thus, we propose that physical activity has
79 a significant effect on sleep quality (Hypothesis 1).

## 80 1.2 The mediating role of self-control

81 The self-control theory and related empirical findings suggest that college students’ self-control may
82 be an important mediating variable of the relationship between physical activity and sleep quality
83 [29]. Self-control refers to an individual’s ability to suppress immediate impulses and regulate his/her
84 behavior to conform to social norms and long-term goals [30]. A strength model shows that self-
85 control, like muscle, can be enhanced through appropriate exercise [31]. Studies in the field of
86 cognitive neuroscience have shown that mild exercise induces cortical activation in the dorsolateral
87 left prefrontal and frontal pole regions and that the activation of these brain regions is associated with
88 self-control increase [32]. Overall, physical activity is significantly positively correlated with both
89 self-control and sleep quality in college students; furthermore, self-control can partially mediate the
90 relationship between physical activity and sleep quality [33]. Physical activity is an exercise pathway
91 that can enhance people’s self-control. Taekwondo training positively affects self-control, self-
92 efficacy, and school-life satisfaction [34]. Studies have also shown that the relationship between self-
93 control and sleep is complex and bidirectional and that there is a significant relationship between
94 physical activity level and self-control. On the one hand, adopting a healthy lifestyle involving
95 regular physical activity and adequate sleep patterns is beneficial to an individual’s self-control [35].
96 On the other hand, metacognitive control over intrusive and unwanted thoughts at bedtime
97 significantly influences sleep quality [36]. Individuals with weak self-control are more likely to have
98 their bedtime delayed by other distractions, thus reducing their sleep duration and resulting in sleep
99 problems such as sleep deprivation and fatigue [37]. Many scholars believe that poor sleep quality
100 and insomnia are closely related to pre-bedtime cognitive arousal (i.e., thought control) [38, 39].
101 Hence, we propose that self-control mediates the relationship between physical activity and sleep
102 quality (Hypothesis 2).

## 103 1.3 The mediating role of mobile phone addiction

104 Mobile phone addiction, also known as “mobile phone dependence” or problematic mobile phone
105 use, is a non-substance addiction. Notably, the non-substance addiction (or behavioral addiction)
106 emcompasses pathological gambling, food addiction, internet addiction, and mobile phone addiction,
107 whereas the substance addiction (or drug addiction) is a neuropsychiatric disorder characterized by a
108 recurring desire to continue taking the drug despite harmful consequences [40]. Mobile phone
109 addiction may be the biggest non-drug addiction in the 21st century (Richard, 2012). Due to the
110 development of Internet technology and the impact of the COVID-19 pandemic, mobile phone
111 addiction among Chinese college students has been on the rise in the past decade (2013-2022) [41].
112 Scholars' attention on mobile phone addiction has shifted away from merely describing this
113 phenomenon towards understanding its detrimental effects while exploring preventive measures [42,
114 43]. During adolescence, cell phone abuse can interfere with healthy activities and habits, especially
115 the duration and quality of sleep. For instance, a Finnish study found that that longer mobile phone
116 use was associated with shorter sleep duration [44]. An Iranian study indicates a significant
117 influential relationship between excessive mobile phone use and general health and sleep quality
118 [45]. A study in India revealed a significant positive correlation between participants’ smartphone
119 addiction and insomnia severity [46].
120 Additionally, some studies show that mobile phone dependence is correlated with physical activity.
121 For example, physical exercise was significantly negatively correlated with mobile phone addiction
122 among college student [47] . Zhong et al. also showed that physical activity has a negative effect on
123 college students’ mobile phone dependence [29]. The Health Action Process Approach model
124 intervention significantly reduced the level of mobile phone addiction among adolescents [48].
125 Accordingly, we propose that mobile phone addiction mediates the relationship between physical
126 activity and sleep quality (Hypothesis 3).

## 127 1.4 Chain mediation of self-control and mobile phone

128 addiction
129 Self-control may play a major linking role in mobile phone addiction, i.e., lower self-control suggests
130 increased social media use, whereas individuals with high self-control are more adept at controlling
131 social media use [49]. One meta-analysis revealed that smartphone addiction in adolescents was
132 strongly negatively correlated with self-control [50]. The inability to control oneself is one of the
133 main causes of all addictive behaviors [51]. Low self-control is often associated with excessive
134 mobile phone use through interpersonal and transactional modeling [52]. In addition, scholars have
135 verified that physical activity variables are negatively correlated with mobile phone dependence
136 variables and positively correlated with self-control variables. They also found that self-control
137 variables are negatively correlated with mobile phone dependence and that self-control partially
138 mediates the effect of physical activity on mobile phone dependence, with the mediating effect
139 accounting for 39.68% of the total effect [53]. Therefore, we propose that self-control and mobile
140 phone addiction chain-mediate the correlations between physical activity and sleep quality
141 (Hypothesis 4).

# 142 2. Research methodology

## 143 2.1 Procedure and participants

144 Participants were recruited from October 8 to October 30, 2023, from different regions of China
145 (Northeast, North, Northwest, East, Central, South, and Southwest). The studies involving human
146 participants were reviewed and approved by the Ethics Committee of Chengdu Sport University
147 (2023-150). The participants were informed that partaking in the study was voluntary and that
148 completing the questionnaire via an online platform indicated their informed consent to voluntarily
149 participate in the study. The participants were to read the research descriptions and consent form and
150 agree to participate, and the survey was designed to end automatically if they did not agree. Once the
151 questionnaire has been submitted, the author has permanent rights to the data. The questionnaire
152 included the purpose and method of research, anonymity and confidentiality regarding participation,
153 and the choice to agree or withdraw from participation, as well as the confirmation that the survey
154 will not be used for purposes other than research. A total of 2274 undergraduates from 34
155 universities were surveyed via the online questionnaire (https:/[/www.wenju](http://www.wenjuan.com/s/UZBZJv98V2/))a[n.com/s/UZBZJv98V2/).](http://www.wenjuan.com/s/UZBZJv98V2/))
156 The participants were 18–24 years old (M = 19.18, SD = 1.02). Females accounted for 67.3% of
157 participants. 45.1% of the subjects used smartphones for over five years. More details were shown in
158 Table **1**. Descriptive data for main variables.
159 Table 1. Descriptive data for main variables

Number

Male, n%	743(32.7%)

Grade

Freshman	28(1.2%)

Sophomore	259(11.4%)

Junior	1349(59.3%)

Senior	638(28.1%)

Age in years, M(SD)	19.18(1.02)

Physical activity, n(%)

Low	1584(69.66%)

Moderate	327(14.38%)

High	363(15.96%)

| Sleep quality, M(SD) | 8.61(3.59) |
| --- | --- |
| Self-control, M(SD) | 2.67(0.61) |
| Mobile phone addition, M(SD) | 3.15(0.93) |

## 160 2.2 Research tools

### 161 2.2.1 Physical Activity Scale

162 Physical activity was assessed using the Physical Activity Rank Scale-3 (Liang and Liu. 1994). This
163 questionnaire is a self-reported physical activity assessment involving data from the previous month
164 through inquiries on intensity, duration, and frequency across five graded levels each. An individual’s
165 total physical activity is calculated as PA = Intensity (1 ~ 5) × Time (0 ~ 4) × Frequency (1 ~ 5),
166 yielding an overall score ranging from 0 to 100. The scores were further classified into low (≤19),
167 moderate (20 ~ 42), or high (≥43). This scale has been widely used in China in recent decades due
168 to the high reliability and validity [54]. The Cronbach’s alpha coefficient for the questionnaire in this
169 study is 0.823.

### 170 2.2.2 Sleep Quality Scale

171 The Chinese version of the Pittsburgh Sleep Quality Index (PSQI) was used to assess subjective sleep
172 quality[55]. The PSQI consists of 7 sections: sleep quality, time to sleep, sleep duration, sleep
173 efficiency, sleep disorders, hypnotic drugs, and daytime dysfunction. Each section is scored from 0
174 and 3. The final score of the PSQI is obtained by cumulatively adding up the scores of each section,
175 and the higher the score, the worse the sleep quality. The Cronbach’s alpha coefficient for the scale is
176 0.911. The reliability and validity of this scale have been reported in the Chinese college student
177 population [56].

### 178 2.2.3 Self-Control Scale

179 The Chinese version of Tangney’s Self-Control Scale (Tan and Guo, 2008) was adopted herein. It
180 was revised according to the characteristics of traditional Chinese culture. It divided self-control into
181 five dimensions, retaining only the nomenclature of the original questionnaire’s three dimensions of
182 impulse control, healthy habits, and concentration on work, while adding two dimensions: resisting
183 temptation and abstaining from entertainment. The scale includes 19 questions. A higher total score
184 indicates better self-control. The scale has shown high reliability and validity among Chinese college
185 students [57]. The Cronbach’s alpha coefficient of the scale is 0.713.

### 186 2.2.4 Mobile Phone Addiction Scale

187 The degree of mobile phone addiction among college students was assessed using the Smartphone
188 addiction scale-short version (SAS-SV) (Kwon et al., 2013), which was developed based on the
189 Korean culture. Given that Korean share a similar demographic structure and background with China,
190 SAS-SV is therefore relatively unaffected by potential cultural differences. The scale consists of 10
191 questions on a 6-point Likert scale (1: “Strongly Disagree”, 6: “Strongly Agree”) and assesses
192 smartphone addiction by self-report. The higher the score, the more severe the mobile phone
193 dependence. The scale shows high reliability and validity in Chinese college students [58]. The
194 Cronbach’s alpha coefficient for this scale was 0.885.

## 195 2.3 Research procedures

196 The data analysis tool is SPSS 26.0 software, which mainly carries out descriptive statistics,
197 confidence tests, common method bias tests, correlation analysis, hypothesis tests, etc. Descriptive
198 statistics are used to understand the basic distribution pattern of the data initially. Confidence tests
199 and common method bias tests are used to ensure that the quality of the questionnaire that meets the
200 requirements of data analysis. The correlation analysis constitutes the premise and basis of regression
201 analysis. Based on the above test results, the regression analysis method is used to infer the statistics
202 of the original hypothesis. The significance test result of p < 0.01 will be used as the critical criterion
203 for the establishment of the hypothesis. In addition, to minimize the error for the test of the mediating
204 effect in the model, the bootstrap method will be used to sample the data 5,000 times to correct any
205 deviations.

# 206 3. Research results

## 207 3.1 Commonly used method deviation tests

208 Given that the data are exclusively derived from questionnaires, there is a potential issue of common
209 method bias arising from the uniform and directional systematic errors in the data results, due to the
210 consistent data collection method. Therefore, it is essential to employ appropriate analytical methods
211 for testing. Typically, Harman’s one-way factor analysis is used to assess common method bias by
212 including all Likert-scale questions in the questionnaire.
213 In this study, SPSS 26.0 was utilized to conduct principal component factor analysis on the data
214 results. The findings revealed that the first-factor interpretation rate of the scale was 24.096%,
215 significantly lower than threshold of 40%, indicating that common method bias was not prominent
216 and our data is applicable to further analysis.

## 217 3.2 Descriptive statistics and correlation analysis for each

218 variable
219 Correlation analysis is a statistical analysis method used to study the correlations between two or
220 more random variables of equal importance, serving as a prerequisite and foundation for regression
221 analysis. The correlation coefficient, typically represented by “r” ranging from -1 to 1, indicates the
222 strength and direction of the correlation. A negative correlation coefficient suggests an inverse
223 relationship between variables, while a positive correlation coefficient signifies a direct relationship.
224 In this study, Pearson correlation analysis was employed to assess the associations among physical
225 activity, self-control, sleep quality, and mobile phone addiction.
226 The correlation coefficient matrix (Table **2**. Descriptive statistics and correlation analysis)
227 reveals significant correlations among the variables. First, physical activity was significantly
228 correlated with sleep quality (r= -0.541, p < 0.001), i.e., a higher value of physical activity is
229 indicative of a lower sleep quality index (i.e., a higher sleep quality), consistent with Hypothesis 1.
230 Second, physical activity was significantly correlated with self-control (r= 0.234, p < 0.001) and
231 mobile phone addiction (r= -0.367, p < 0.001), respectively. Third, sleep quality was also
232 significantly correlated with self-control (r= -0.540, p < 0.001) and mobile phone addiction (r= 0.572,
233 p < 0.001), respectively. This correlation analysis suggests that both self-control and mobile phone
234 addiction affect the correlations between physical activity and sleep quality. Notably, there is a
235 positive correlation between sleep quality and mobile phone addiction, i.e., the higher the mobile
236 phone addiction, the higher the sleep quality index score and the worse the sleep quality. These
237 results satisfy the prerequisites for regression analysis.
238 Table 2. Descriptive statistics and correlation analysis

M	SD

Physical activity

Self-control

Mobile	phone addiction

Sleep quality

| Physical activity 18.80 | 21.20 | 1 |  |  |
| --- | --- | --- | --- | --- |
| Self-control 63.28 | 11.57 | 0.234*** | 1 |  |
| Mobile phone  31.50 | 9.32 | -0.367*** | -0.414*** | 1 |
| addiction |  |  |  |  |
| Sleep quality 8.61 | 3.59 | -0.541*** | -0.540*** | 0.572*** 1 |

239 ****p<0.001, **p<0.01, *p<0.05*

## 240 3.3 Relationship between physical activity and sleep quality:

241 A chain mediation model
242 Based on the above correlation analysis, we constructed a chain mediation effect model (Fig **1** The
243 chain mediation model (*** p < 0.001)) with physical activity as the independent variable, sleep
244 quality as the dependent variable, and self-control and mobile phone addiction as mediator variables.
245 The stepwise regression analysis was employed to examine the influence of physical activity on the
246 sleep quality. Furthermore, we conducted an assessment to determine whether there existed a
247 significant chain mediation effect of self-control and mobile phone addiction in this relationship.
248 Fig 1 The chain mediation model (*** p < 0.001)
249 In the variable regression relationship test, the covariance VIF of physical activity, self-control, and
250 mobile phone addiction variables were 1.167, 1.218, and 1.330, respectively. All of these values were
251 below 2, suggesting that there were no serious covariance problems between the variables. Physical
252 activity had a significant positive regression effect on self-control (standardized regression
253 coefficient β = 0.234, p < 0.001), a significant negative regression effect on mobile phone addiction
254 (β = –0.286, p < 0.001), and a significant negative regression effect on sleep quality (β = –0.351, p <
255 0.001). Self-control showed a significant negative regression effect on mobile phone addiction (β=-
256 0.347, p<0.001), and a significant negative regression effect on sleep quality (β = –0.331, p < 0 .001).
257 Mobile phone addiction had a significant positive regression effect on sleep quality (β = 0.306, p <
258 0.001) (Table **3**.Regression coefficient test). Therefore, the relationship between the regression
259 effects of each variable in the model was verified.
260 Table 3.Regression coefficient test

| Outcome  variable | Predictor variable | R | R2 | F | β | t |
| --- | --- | --- | --- | --- | --- | --- |
| Self-control | Constants  physical activity | 0.234 | 0.055 | 132.171*** | 2.796***  0.234*** | 168.471  11.497 |
|  | Constants |  |  |  | 1.969*** | 23.655 |
| Mobile phone  addiction | physical activity | 0.498 | 0.248 | 375.137*** | -0.286*** | -15.268 |
|  | self-control |  |  |  | -0.347*** | -18.528 |
|  | Constants |  |  |  | 0.800** | 2.871 |
|  | physical activity |  |  |  | -0.351*** | -22.916 |
| Sleep quality | self-control | 0.738 | 0.544 | 903.234*** | -0.331*** | -21.188 |

261

mobile	phone addiction

****p < 0.001, **p < 0.01, *p < 0.05*

0.306***	18.744

262 The process function was used to calculate the amount of each mediating effect in the model. To
263 minimize the error, each parameter was sampled 5000 times using the bootstrap method for bias
264 correction. According to the calculation results (Table **4**. Bootstrap test for mediating effects), the
265 total effect of physical activity on sleep quality in the model is –0.541. The total indirect effect
266 amount is –0.190, accounting for 35.16% of the total effect, with the 99% confidence interval of [–
267 0.212, –0.168]. There were three indirect effects: self-control, mobile phone addiction, and self-
268 control & mobile phone addition. They accounted for 14.36%, 16.18%, and 4.60% of the total
269 effects, respectively. Their confidence interval (99%) is [–0.212, –0.168], [–0.091, –0.063], and [–
270 0.101, –0.075], respectively.
271 Table 4. Bootstrap test for mediating effects

| Effect | | | BOOT SE | BOOT LLCI | Relative mediation  BOOT ULCI | |
| --- | --- | --- | --- | --- | --- | --- |
|  | | |  |  | effect | |
| Total effect –0.541 | | | 0.018 | –0.576 | –0.506 |  |
| Direct effect –0.351 | | | 0.015 | –0.381 | –0.321 |  |
| Total indirect  –0.190 | | | 0.011 | –0.212 | –0.168 | 35.16% |
| effect | | |  |  |  |  |
| Indirect effect 1  –0.078 | | | 0.007 | –0.091 | –0.063 | 14.36% |
| (self-control)  Indirect effect 2 | | |  |  |  |  |
| (mobile | phone | –0.088 | 0.007 | –0.101 | –0.075 | 16.18% |
| addiction) |  |  |  |  |  |  |
| Indirect effect 3 (self-control & | | | | | | |
| –0.025 | | | 0.003 | –0.031 | –0.019 | 4.60% |

mobile	phone addiction)

272 *Indirect effect 1: physical activity* → *self-control* → *sleep quality;*
273 *Indirect effect 2: physical activity* → *mobile phone addiction* → *sleep quality;*
274 *Indirect effect 3: physical activity* → *self-control* → *mobile phone addiction* → *sleep quality.*

# 275 4. Discussion

276 First, this study shows that physical activity levels were negatively correlated with college students’
277 sleep quality index. The PSQI scale scoring rules indicate that the higher the PSQI score, the poorer
278 the quality of sleep; thus, the higher the physical activity level, the better the sleep quality (i.e., the
279 lower the frequency of sleep disorders). This study validated Hypothesis 1, consistent with previous
280 findings [33, 59]. A meta-analysis showed that scientific and reasonable physical activity can improve
281 sleep quality [60]. Students with high levels of physical activity have longer nighttime sleep, fewer
282 awakenings, and better sleep quality [61]. A cross-sectional study also shows that college students who
283 are physically active enough also have better sleep quality [7, 62]. This implies that participation in
284 various types of physical activities effectively promotes sleep quality and reduces sleep disorders.
285 However, some studies have also shown that physical activity does not have a significant effect on
286 sleep quality in young people [17], and that high-intensity physical activity at night has no significant
287 effect on sleep quality [63]. The intensity of physical activity adopted and the duration of physical
288 activity performed to enhance sleep quality warrant further in-depth exploration in future.
289 Second, this study showed that self-control mediates the relationship between physical activity and
290 sleep quality, and thus Hypothesis 2 was validated. Physical activity significantly and positively
291 predicted self-control, while self-control negatively predicted sleep quality, i.e., the higher the level of
292 physical activity, the greater the self-control, the lower the PSQI score, and the better the sleep quality.
293 This is consistent with previous findings [64]. An intervention study has found that sleep-related self-
294 regulation techniques can reduce poor sleep habits and enhance the quality of sleep [65]. A meta-
295 analysis showed modest positive associations between self-control and physical activity, healthier
296 eating and healthier sleep [66]. Correlational meta-analytic studies have shown that acute exercise at
297 multiple intensities can enhance individuals’ executive functioning [67]. Cognitive neuroscience
298 studies have also determined that mild exercise induces cortical activation in the dorsolateral left
299 prefrontal and frontal pole regions, and that the activation of these brain regions is associated with
300 increased self-control [32]. This better explains the role of self-control as a mediator between physical
301 activity and sleep quality. However, it has also been proposed that better quality and quantity of sleep
302 is influenced by factors outside of self-control, such as environment [68] and individual differences
303 [69].
304 Third, this study suggests that mobile phone addiction has a mediating effect on the correlations
305 between physical activity and sleep quality. As the level of physical activity increases, the mobile phone
306 dependence index and sleep quality index decrease, and sleep quality improves. The results of this
307 study consolidate Hypothesis 3 and agree with previous findings [70]. According to immersion theory,
308 when college students are immersed in mobile phones, their endocrine systems are in a state of
309 continuous activity, gradually releasing adrenaline, dopamine, and other excitatory hormones that keep
310 the body in a state of excitement, leading to disruption in life patterns, which in turn affects the quality
311 of sleep [71, 72]. In addition, mobile phone addiction causes college students to spend a significant
312 amount of time playing games on their mobile phones, which reduces the amount of time spent in
313 sleeping and exercising, which in turn affects the quality of their sleep [73]. Therefore, controlling the
314 time spent on mobile phones to reduce mobile phone addiction and increase opportunities for physical
315 activity among college students can help improve the quality of sleep among college students. While
316 some studies have shown that physical activity can reduce mobile phone use, others have pointed out
317 that in some cases, physical activity may increase reliance on mobile phones, especially when
318 individuals use them to record exercise data or share exercise achievements [74].
319 Finally, this study indicates that self-control and mobile phone addiction are closely related. They
320 constitute intermediate links in the pathway: physical activity → self-control → mobile phone
321 addiction → sleep quality influence, and there is a chain-mediated effect in the process of physical
322 activity influencing sleep quality. Specifically, college students who score higher on physical activity
323 tend to have lower PSQI scores, indicative of better sleep quality, due to their greater self-control and
324 reduced phone addiction. A strong correlation is observed between self-control and mobile phone
325 addiction, i.e., higher levels of self-control are associated with lower mobile phone dependence and
326 vice versa. However, the effect sizes of chained mediators were not as high as those of mediators alone,
327 probably due to the negative correlation between self-control and mobile phone addiction, and thus
328 their interaction may weaken the effect of chained mediators when both are used together as mediating
329 variables. This finding supports Hypothesis 4 and aligns with previous studies [75, 76]. The present
330 study confirms that self-control and mobile phone addiction have chain-mediated effects on the process
331 of physical activity, affecting college students’ sleep quality. In addition, another study highlights the
332 role of anxiety and depression as a chain mediator in the relationship between physical activity and
333 sleep quality [77]. It has also been shown that addiction to short videos directly affects the sleep quality
334 of college students, indirectly through physical activity and procrastination behaviors [78]. Thus,
335 complex interactions exist between these variables, necessitating further investigation.
336 Overall, physical activity has a positive effect on promoting college students’ levels of self-control and
337 improving mobile phone dependence. Through self-control, mobile phone dependence can be
338 effectively contained, thereby improving sleep quality. At the same time, improving physical activity
339 levels also helps reduce college students’ mobile phone dependence. Therefore, the chain mediation
340 model of physical activity → self-control → mobile phone addiction → sleep quality proposed in
341 this study is feasible.
342 This study, however, is not without its limitations. First, our study was cross-sectional, and its
343 predictions failed to reveal the causal relationships between physical activity and sleep quality. Second,
344 the questionnaire we used to measure college students’ physical activity level, self-control level,
345 mobile phone dependence index, and sleep quality was subjective. Therefore, future research should
346 utilize technological tools and combine experimental and follow-up research methods to improve the
347 objectivity of data measurement and explain the relationship between physical activity and sleep
348 quality scientifically and reasonably.

# 349 5. Conclusion

350 In this study, we explored the mechanism underlying how physical activity influences college students
351 ’ sleep quality through a cross-sectional survey study. We proposed a chain mediation model: physical
352 activity → self-control → mobile phone addiction → sleep quality, which provides ideas for
353 college students to improve their sleep quality. The results of this study offer valuable insights and
354 serve as a reference for future investigations into the mechanisms through which physical activity

355

356

357

358

influences the enhancement of sleep quality in college students.

359

| 360 | Supporting information |
| --- | --- |
| 361 | S1 Data. |
| 362 | (xlsx) |
| 363 |  |

# 364 References

365 1.	Worley SL. The Extraordinary Importance of Sleep: The Detrimental Effects of Inadequate Sleep
366 on Health and Public Safety Drive an Explosion of Sleep Research. P & T : a peer-reviewed
367 journal for formulary management. 2018;43(12):758-63.
368 2.	Pavlova MK, Latreille V. Sleep Disorders. American Journal of Medicine. 2019;132(3):292-9.
369 3.	Koohsari MJ, Yasunaga A, McCormack GR, Shibata A, Ishii K, Liao Y, et al. Sedentary
370 behaviour and sleep quality. Scientific Reports. 2023;13(1).
371 4.	Cao XL, Wang SB, Zhong BL, Zhang L, Ungvari GS, Ng CH, et al. The prevalence of insomnia
372 in the general population in China: A meta-analysis. PloS one. 2017;12(2).
373 5.	Claydon EA, Kahwash JM, Lilly CL, Alamir Y, Zullig KJ. Subjective Sleep Quality, Caffeine,
374 and Dieting Behaviors Among University-Attending Young Adults. Nature and Science of Sleep.
375 2023;15:737-47.
376 6.	Kumar S, Rajasegaran R, Prabhakaran S, Mani T. Extent of Smartphone Addiction and its
377 Association with Physical Activity Level, Anthropometric Indices, and Quality of Sleep in
378 Young Adults: A Cross-Sectional Study. Indian Journal of Community Medicine.
379 2024;49(1):199-202.
380 7.	Wang F, Biro E. Determinants of sleep quality in college students: A literature review. Explore-
381 the Journal of Science and Healing. 2021;17(2):170-7.
382 8.	Li Y, Bai W, Zhu B, Duan R, Yu X, Xu W, et al. Prevalence and correlates of poor sleep quality
383 among college students: a cross-sectional survey. Health and Quality of Life Outcomes.
384 2020;18(1).
385 9.	Zhou Y, Bo S, Ruan S, Dai Q, Tian Y, Shi X. Deteriorated sleep quality and influencing factors
386 among undergraduates in northern Guizhou, China. Peerj. 2022;10.
387 10. Hu B, Shen W, Wang Y, Wu Q, Li JL, Xu XZ, et al. Prevalence and related factors of sleep
388 quality among Chinese undergraduates in Jiangsu Province: multiple models' analysis. Frontiers
389 in psychology. 2024;15.
390 11. Ramos-Munell J, Pozo-Cruz J, Alfonso-Rosa RM, Alvarez-Barbosa F. Effects of a Physical
391 Activity Intervention in the School Setting on Sleep Quality and Quantity in Preschoolers: A
392 Systematic Review. Retos-Nuevas Tendencias En Educacion Fisica Deporte Y Recreacion.
393 2024(51):235-42.
394 12. Chrysant SG. Effects of physical activity on sleep quality and wellbeing. Hospital practice

395	(1995). 2024;52(1-2):13-8.

396 13. Master L, Nye RT, Lee S, Nahmod NG, Mariani S, Hale L, et al. Bidirectional, Daily Temporal
397 Associations between Sleep and Physical Activity in Adolescents. Scientific Reports. 2019;9.
398 14. Hartescu I, Morgan K, Stevinson CD. Increased physical activity improves sleep and mood
399 outcomes in inactive people with insomnia: a randomized controlled trial. Journal of Sleep
400 Research. 2015;24(5):526-34.
401 15. Fukuie T, Inoue K, Yamaguchi A. Lifestyle Elements for Improving Mental and Physical Health
402 in Japanese University Students: Subjective Sleep Quality is a Common Key Factor. American
403 Journal of Lifestyle Medicine. 2023.
404 16. Rosa CC, Tebar WR, Soares Oliveira CB, Farah BQ, Casonatto J, Ciccotti Saraiva BT, et al.
405 Effect of Different Sports Practice on Sleep Quality and Quality of Life in Children and
406 Adolescents: Randomized Clinical Trial. Sports Medicine-Open. 2021;7(1).
407 17. Zhao H, Lu C, Yi C. Physical Activity and Sleep Quality Association in Different Populations: A
408 Meta-Analysis. International journal of environmental research and public health. 2023;20(3).
409 18. Grandner MA, Fernandez F-X. The translational neuroscience of sleep: A contextual framework.

410	Science. 2021;374(6567):568-73.

411	19. Nelson KL, Davis JE, Corbett CF. Sleep quality: An evolutionary concept analysis. Nursing

412	Forum. 2022;57(1):144-51.

413	20. Che T, Yan C, Tian D, Zhang X, Liu X, Wu Z. The Association Between Sleep and Metabolic

414	Syndrome: A Systematic Review and Meta-Analysis. Frontiers in Endocrinology. 2021;12.

415	21. Peter-Derex L, Yammine P, Bastuji H, Croisile B. Sleep and Alzheimer's disease. Sleep Medicine

416	Reviews. 2015;19:29-38.

417 22. Schmickler JM, Blaschke S, Robbins R, Mess F. Determinants of Sleep Quality: A Cross-
418 Sectional Study in University Students. International journal of environmental research and
419 public health. 2023;20(3).
420 23. Ma X, Meng D, Zhu L, Xu H, Guo J, Yang L, et al. Bedtime procrastination predicts the
421 prevalence and severity of poor sleep quality of Chinese undergraduate students. Journal of
422 American College Health. 2022;70(4):1104-11.
423 24. Wang F, Boros S. The effect of physical activity on sleep quality: a systematic review. European
424 Journal of Physiotherapy. 2021;23(1):11-8.
425 25. Alnawwar MA, Alraddadi MI, Algethmi RA, Salem GA, Salem MA, Alharbi AA. The Effect of
426 Physical Activity on Sleep Quality and Sleep Disorder: A Systematic Review. Cureus Journal of
427 Medical Science. 2023;15(8).
428 26. Ferreira MF, Bos SC, Macedo AF. The impact of physical activity on objective sleep of people
429 with insomnia. Psychiatry Research. 2023;320.
430 27. Mochon-Benguigui S, Carneiro-Barrera A, Castillo MJ, Amaro-Gahete FJ. Role of physical
431 activity and fitness on sleep in sedentary middle-aged adults: the FIT-AGEING study. Scientific
432 Reports. 2021;11(1).
433 28. Khazaie H, Norouzi E, Rezaie L, Safari-Faramani R. Effect of physical activity on sleep quality
434 in patients with major depression disorder: A systematic review and meta-analysis of randomized
435 controlled trials. Current Psychology. 2023;42(33):28846-56.
436 29. Zhong W, Wang Y, Zhang G. The Impact of Physical Activity on College Students’ Mobile
437 Phone Dependence: the Mediating Role of Self-Control. International Journal of Mental Health
438 and Addiction. 2021;19(6):2144-59.
439 30. Meldrum RC, Barnes JC, Hay C. Sleep Deprivation, Low Self-Control, and Delinquency: A Test
440 of the Strength Model of Self-Control. Journal of Youth and Adolescence. 2015;44(2):465-77.
441 31. Baumeister RF, Vohs KD, Tice DM. The strength model of self-control. Current Directions in
442 Psychological Science. 2007;16(6):351-5.
443 32. Byun K, Hyodo K, Suwabe K, Ochi G, Sakairi Y, Kato M, et al. Positive effect of acute mild
444 exercise on executive function via arousal-related prefrontal activations: An fNIRS study.
445 Neuroimage. 2014;98:336-45.
446 33. Ye J, Jia X, Zhang J, Guo K. Effect of physical exercise on sleep quality of college students:
447 Chain intermediary effect of mindfulness and ruminative thinking. Frontiers in psychology.
448 2022;13.
449 34. Keun-Woo S, Sung-Il K, Hwang，Young S. The Effects of Teenage Taekwondo Training on
450 Self-control, Self-efficacy and School Life Satisfaction. The Korean Society of Sports Science.
451 2019;28(4):807-18.
452 35. Li L, Yu Q, Zhao W, Herold F, Cheval B, Kong Z, et al. Physical Activity and Inhibitory Control:
453 The Mediating Role of Sleep Quality and Sleep Efficiency. Brain Sciences. 2021;11(5).
454 36. Sella E, Borella E. Strategies for controlling sleep-related intrusive thoughts, and subjective and
455 objective sleep quality: how self-reported poor and good sleepers differ. Aging & Mental Health.
456 2021;25(10):1959-66.
457 37. Kroese FM, Evers C, Adriaanse MA, de Ridder DTD. Bedtime procrastination: A self-regulation
458 perspective on sleep insufficiency in the general population. Journal of Health Psychology.
459 2016;21(5):853-62.
460 38. Ree MJ, Harvey AG, Blake R, Tang NKY, Shawe-Taylor M. Attempts to control unwanted
461 thoughts in the night: development of the thought control questionnaire-insomnia revised
462 (TCQI-R). Behaviour Research and Therapy. 2005;43(8):985-98.
463 39. Capkova K, Vaculik M, Ellis J, Sipula M. The impact of pre-sleep arousal state and strategy to
464 control unwanted thoughts on sleep quality. Anxiety Stress and Coping. 2018;31(3):338-47.
465 40. Zou Z, Wang H, Uquillas FdO, Wang X, Ding J, Chen H. Definition of Substance and Non-
466 substance Addiction. In: Zhang X, Shi J, Tao R, editors. Substance and Non-Substance
467 Addiction. Advances in Experimental Medicine and Biology. 10102017. p. 21-41.
468 41. Lyu C, Cao Z, Jiao Z. Changes in Chinese college students’ mobile phone addiction over recent
469 decade: The perspective of cross-temporal meta-analysis. Heliyon. 2024;10(11):e32327.
470 42. Liu FB, Xi Y, Li N, Wu M. Brief Mindfulness Training Mitigates College Students' Mobile
471 Phone Addiction: The Mediating Effect of the Sense of Meaning in Life. Psychology research
472 and behavior management. 2024;17:273-82.
473 43. Tao YQ, Tang QH, Wang SJ, Zou XY, Ma ZJ, Zhang L, et al. The impact of long-term online
474 learning on social anxiety and problematic smartphone use symptoms among secondary school
475 students with different levels of fear of missing out: Evidence from a symptom network and
476 longitudinal panel network analysis. Journal of Behavioral Addictions. 2024;13(1):102-19.
477 44. Cain N, Gradisar M. Electronic media use and sleep in school-aged children and adolescents: A

478	review. Sleep Medicine. 2010;11(8):735-42.

479 45. Eyvazlou M, Zarei E, Rahimi A, Abazari M. Association between overuse of mobile phones on
480 quality of sleep and general health among occupational health and safety students.
481 Chronobiology International. 2016;33(3):293-300.
482 46. Telgote SA, Ghogare AS, Khadse V, Karwande SG. Smartphone Addiction and its Impact on
483 Insomnia among the Undergraduate Medical Students of a Teaching Hospital of Maharashtra,
484 India-A Cross-sectional study. Journal of Clinical and Diagnostic Research. 2021;15(12):VC1-
485 VC5.
486 47. Tong W, Meng S. Effects of Physical Activity on Mobile Phone Addiction Among College
487 Students: The Chain-Based Mediating Role of Negative Emotion and E-Health Literacy.
488 Psychology research and behavior management. 2023;16(null):3647-57.
489 48. Zhang L, Chen X, Zhu L. Effect of an Intervention Based on the Health Action Process
490 Approach Model on Mobile Phone Addiction in Adolescents. Iranian Journal of Public Health.
491 2024;53(6):1284-92.
492 49. Brevers D, Turel O. Strategies for self-controlling social media use: Classification and role in
493 preventing social media addiction symptoms. Journal of Behavioral Addictions. 2019;8(3):554-
494 63.
495 50. Ding Y, Wan X, Lu G, Huang H, Liang Y, Yu J, et al. The associations between smartphone
496 addiction and self-esteem, self-control, and social support among Chinese adolescents: A meta-
497 analysis. Frontiers in psychology. 2022;13.
498 51. Bhargava VR, Velasquez M. Ethics of the Attention Economy: The Problem of Social Media
499 Addiction. Business Ethics Quarterly. 2021;31(3):321-59.
500 52. Servidio R. Self-control and problematic smartphone use among Italian University students: The
501 mediating role of the fear of missing out and of smartphone use patterns. Current Psychology.
502 2021;40(8):4101-11.
503 53. Zhang D, Su T, Lyu B, Yang Y, Zhuo X. The effects of adolescent physical activity participation
504 on cell phone dependence: The mediating role of self-control. Work-a Journal of Prevention
505 Assessment & Rehabilitation. 2022;72(4):1289-98.
506 54. Feng, Y. J., Mao, Z. X., and Che, G. W. (2015). Construction of intervention model for
507 predicting physical activity behavior of junior middle school students: Combination of automatic
508 machine and TPB extended model. Journal of Beijing Sport University. 38, 72–76. doi:
509 10.19582/j.cnki.11-3785/g8.2015.05.014
510 55. Liu XC, Tang MQJCJoP. Reliability and validity of the Pittsburgh Sleep Quality Index.

511	1996;29(2):29103-7.

512 56. Liu X, Lang L, Wang R, Chen W, Ren X, Lin Y, et al. Poor sleep quality and its related risk
513 factors among university students. 2021. 2021;10(4):4479-85.
514 57. Jiang ZC, Zhao XX. Self-control and problematic mobile phone use in Chinese college students:
515 the mediating role of mobile phone use patterns. Bmc Psychiatry. 2016;16.
516 58. Zhao H, Rafik-Galea S, Fitriana M, Song TJ. Translation and psychometric evaluation of
517 Smartphone Addiction Scale-Short Version (SAS-SV) among Chinese college students. PloS
518 one. 2022;17(11).
519 59. Li Y, Guo K. Research on the relationship between physical activity, sleep quality, psychological
520 resilience, and social adaptation among Chinese college students: A cross-sectional study.
521 2023;14.
522 60. Kredlow MA, Capozzoli MC, Hearon BA, Calkins AW, Otto MW. The effects of physical
523 activity on sleep: a meta-analytic review. Journal of Behavioral Medicine. 2015;38(3):427-49.
524 61. Lang C, Brand S, Feldmeth AK, Holsboer-Trachsler E, Pühse U, Gerber M. Increased self-
525 reported and objectively assessed physical activity predict sleep quality among adolescents.
526 Physiology & Behavior. 2013;120:46-53.
527 62. Stefan L, Kristicevic T, Sporis G. The associations of self-reported physical fitness and physical
528 activity with sleep quality in young adults: A population-based study. Mental Health and
529 Physical Activity. 2018;14:131-5.
530 63. F OR, Maitha A, Moza A, Aisha A, Mariam A, Amna A. Morning Versus Evening Physical
531 Activity, Sleep Quality, and Psychological Well-Being Among Healthy Adults in the UAE. sleep
532 medicine research. 2024;15(1):59-66.
533 64. Zhong W, Wang Y, Zhang G. The Impact of Physical Activity on College Students' Mobile
534 Phone Dependence: the Mediating Role of Self-Control. International Journal of Mental Health
535 and Addiction. 2021;19(6):2144-59.
536 65. Loft MH, Cameron LD. Using Mental Imagery to Deliver Self-Regulation Techniques to
537 Improve Sleep Behaviors. Annals of Behavioral Medicine. 2013;46(3):260-72.
538 66. Andrade FC, Hoyle RH. A synthesis and meta-analysis of the relationship between trait self-
539 control and healthier practices in physical activity, eating, and sleep domains. Personality and
540 Individual Differences. 2023;205.
541 67. Chang YK, Labban JD, Gapin JI, Etnier JL. The effects of acute exercise on cognitive
542 performance: A meta-analysis. Brain Research. 2012;1453:87-101.
543 68. Gao C, Chapagain NY, Scullin MK. Sleep Duration and Sleep Quality in Caregivers of Patients
544 With Dementia A Systematic Review and Meta-analysis. Jama Network Open. 2019;2(8).
545 69. Duggan KA, Friedman HS, McDevitt EA, Mednick SC. Personality and Healthy Sleep: The
546 Importance of Conscientiousness and Neuroticism. PloS one. 2014;9(3).
547 70. Xu C-Y, Zhu K-T, Ruan X-y, Zhu X-Y, Zhang Y-S, Tong W-X, et al. Effect of physical exercise
548 on sleep quality in college students: Mediating role of smartphone use. PloS one. 2023;18(11).
549 71. Parekh PK, McClung CA. Circadian Mechanisms Underlying Reward-Related Neurophysiology
550 and Synaptic Plasticity. Frontiers in Psychiatry. 2016;6.
551 72. Jensen JL, Jones CR, Kartsonaki C, Packer KA, Adler FR, Liou TG. Sleep Phase Delay in Cystic
552 Fibrosis A Potential New Manifestation of Cystic Fibrosis Transmembrane Regulator
553 Dysfunction. Chest. 2017;152(2):386-93.
554 73. Mahapatra S. Smartphone addiction and associated consequences: role of loneliness and self-
555 regulation. Behaviour & Information Technology. 2019;38(8):833-44.
556 74. Rodriguez-Larrad A, Manas A, Labayen I, Gonzalez-Gross M, Espin A, Aznar S, et al. Impact of
557 COVID-19 Confinement on Physical Activity and Sedentary Behaviour in Spanish University
558 Students: Role of Gender. International journal of environmental research and public health.
559 2021;18(2).
560 75. Han L, Geng J, Jou M, Gao F, Yang H. Relationship between shyness and mobile phone
561 addiction in Chinese young adults: Mediating roles of self-control and attachment anxiety.
562 Computers in Human Behavior. 2017;76:363-71.
563 76. Xiang M-Q, Lin L, Wang Z-R, Li J, Xu Z, Hu M. Sedentary Behavior and Problematic
564 Smartphone Use in Chinese Adolescents: The Moderating Role of Self-Control. Frontiers in
565 psychology. 2020;10.
566 77. Su Y, Wang S-B, Zheng H, Tan W-Y, Li X, Huang Z-H, et al. The role of anxiety and depression
567 in the relationship between physical activity and sleep quality: A serial multiple mediation
568 model. Journal of Affective Disorders. 2021;290:219-26.
569 78. Zhao Z, Kou Y. Effect of short video addiction on the sleep quality of college students: chain
570 intermediary effects of physical activity and procrastination behavior. Frontiers in psychology.
571 2024;14.

572

Figure 1	[Click here to access/download;Figure;Figure 1.jpg](https://www2.cloud.editorialmanager.com/pone/download.aspx?id=37226409&guid=7de95572-c7e7-4e7f-91e2-ff70a4658ef3&scheme=1)[<https://www2.cloud.editorialmanager.com/pone/download.aspx?id=37226409&guid=7de95572-c7e7-4e7f-91e2-ff70a4658ef3&scheme=1>](https://www2.cloud.editorialmanager.com/pone/download.aspx?id=37226409&guid=7de95572-c7e7-4e7f-91e2-ff70a4658ef3&scheme=1)

Supporting Information

[Click here to access/download](https://www2.cloud.editorialmanager.com/pone/download.aspx?id=37240014&guid=ad8a4560-fa67-4ded-a3d4-d42100c573b1&scheme=1)

[**Supporting Information** S1 Data.xlsx](https://www2.cloud.editorialmanager.com/pone/download.aspx?id=37240014&guid=ad8a4560-fa67-4ded-a3d4-d42100c573b1&scheme=1)
