# Supplementary material for: The relationship between physical activity and sleep quality among college students: The chain-mediating effects of self-control and mobile phone addiction
Source: PLoS One. 2024 Dec 19;19(12):e0315930. doi: 10.1371/journal.pone.0315930 (PMC11658481; doi:10.1371/journal.pone.0315930)
Supplement: Attachment — Submitted filename: Response to Reviewers.docx [file pone.0315930.s003.docx]

Response letter

Dear Dr. Mohammad Sidiq and Reviewers:

On behalf of my co-authors, we sincerely appreciate you and the reviewers for your precious time in reviewing our paper and give us another chance to revise our manuscript. It was your valuable and insightful comments that led to possible improvements in the revised paper. According to you and the reviewers’ comments, we have carefully considered the comments and tried our best to address every one of them. We hope the manuscript after careful revisions can meet your high standards and expectations. Below we provide the point-by-point responses, all modifications in the manuscript have been highlighted in yellow.

Once again, we would like to express our great appreciation to you and the reviewers for your warm work and comments on our paper. We look forward to hearing from you!

Thank you and best regards,

Chengbo Yang, Ph.D.

Corresponding author:

Name: Chengbo Yang

E-mail: yangchengbo2024@sina.com

**Response to Editor**

Dear Editor:

Thank you for your timely follow-up during the review process of this paper, we sincerely appreciate for your dedicated and valuable time! It is your valuable and precious comments that helped greatly in improving our manuscript. After studying your comments carefully, we have dedicated a lot of time and major efforts and tried our best to revise the manuscript. We sincerely hope that the revised manuscript can meets your expectations. Once again, thank you very much for your comments and suggestions. Below we have provided a point-by-point response to your valuable comments.

**1. Introduction:**

***Comment #1. Explain why the chain-mediation model is critical to the discussion early in the article. Discuss the significance of examining self-control as well as the mobile phone addiction as the mediators, specifically in the light of contemporary students’ life.***

**Response:** Thank you for your precious comment. Based on your comments, in the introduction section, we discuss the significance of examining self-control and the collection of addictions as a mediator of physical activity's impact on sleep quality in the context of the information age environment faced by contemporary college students. The specific changed content is as follows.

**[Pg3, Ln21]- [Pg4, Ln10]: “**With the ubiquity of electronic devices in contemporary society, the influence of mobile phone use on the sleep quality of college students has become a significant concern. It is posited that enhancing individual self-regulation can mitigate the risk of mobile phone addiction [25, 26]. Furthermore, engaging in regular physical activity has been shown to bolster self-regulation [27, 28]. This raises the question of whether the positive effect of physical activity on sleep quality is mediated by a sequential process involving the enhancement of self-regulation and the reduction of mobile phone addiction. This hypothesis warrants empirical investigation as a promising research direction. Therefore, we undertook a cross-sectional study focusing on university students in China to delve into the mechanisms that connect physical activity and sleep quality among young individuals. The findings of our study contribute to a broader understanding of the interplay between physical activity and sleep quality.**”**

***Comment #2. Broaden the global relevance: In a larger part, the introduction is dedicated to Chinese students, or rather their experiences and perceptions of living with the respondents. To widen the scope, it is only suitable to briefly explain how the conclusions given might apply to college students in other areas.***

**Response:** Thank you for your precious comment. Based on your comments, we believe that the insights gained from this study, as well as interventions to improve sleep quality and promote physical and mental health, may be applicable in other regions, taking into account different environmental and cultural factors. The specific changed content is as follows.

**[Pg4, Ln10]- [Pg4, Ln16]: “**Moreover, the insights derived from this research may extend beyond the immediate context, offering valuable guidance for the development of interventions aimed at enhancing sleep quality and fostering physical and mental well-being. While our study was centered on Chinese university students, the identified relationships and potential interventions could be relevant and adaptable for college students in other geographic locations, provided that cultural and environmental factors are taken into consideration in the translation of these findings to different settings.**”**

**2. Methods:**

***Comment #3.*** ***Improve the understanding of the measures of the main constructs such as self-control, physical activity, and mobile phone addiction. Make it easier and shorter for the readers especially when it comes to the particular scales used in the research.***

**Response:** Thank you for your precious comment. Based on your comments, and in order to make it easier for readers to understand the scales we use, we have made simpler descriptions and removed some of the less understandable elements. The specific changed content is as follows.

**[Pg11, Ln25]- [Pg12, Ln3]: “**The scale assesses physical activity levels in terms of intensity, frequency, and duration, employing a 5-point Likert scale. The exercise amount is calculated using the formula 'Exercise amount = Exercise frequency * (Exercise duration in minutes - 1) * Exercise intensity,' where the highest possible score is 100 and the lowest possible score is 0, the higher the score, the higher the physical activity level.**”**

**[Pg12, Ln16]- [Pg12, Ln19]: “**The scale consists of five dimensions, Resisting Temptation, Healthy Habits, Abstaining from Entertainment, Impulse Control, and Focused Work, and is based on a 5-point Likert scale.**”**

**[Pg12, Ln24]- [Pg13, Ln2]:** “The scale consists of 10 questions on a 6-point Likert scale (1: “Strongly Disagree”, 6: “Strongly Agree”) and assesses smartphone addiction by self-report. The higher the score, the more severe the mobile phone dependence.”

***Comment #4. It is about time to explain the limitations of self-reported data more elaborately. Although this is done, more detailed consideration of response bias especially in an online survey could have been done in a more detailed manner thus enhancing the credibility of the methodology.***

**Response:** Thank you for your precious comment. Based on your comments, we review the work done to reduce response bias in the design of the questionnaire and its distribution. This was supplemented with additional explanations. The specific changed content is as follows.

**[Pg10, Ln20]- [Pg11, Ln1]: “**To mitigate response bias in the online questionnaire, several measures were implemented. Respondents were restricted to one submission per IP address to prevent multiple entries. Additionally, we employed a one-question-per-page format to enhance focus and reduce cognitive load. We assured respondents of the anonymity and confidentiality of their responses to minimize social desirability bias.**”**

***Comment #5. Consider inclusion of objective measures: One of the future directions for the study is to employ more objective measures for physical activity using pedometers or more objectively measure sleep quality using*** ***actigraphs.***

**Response:** Thank you for your precious comment. Based on your comments, we have added the previously set inclusion criteria. The discussion in our future research directions suggests that the use of pedometers and actigraphs would be one of the more objective and accurate methods for measurement. The specific changed content is as follows.

**[Pg10, Ln16]- [Pg10, Ln20]: “**The questionnaire included the purpose and method of research, anonymity and confidentiality regarding participation, and the choice to agree or withdraw from participation, participant requirements (1. enrolled students, 2. no physical disabilities, 3. no mental illness), as well as the confirmation that the survey will not be used for purposes other than research.**”**

**[Pg24, Ln8]- [Pg24, Ln12]: “**Consequently, a promising direction for future research involves utilizing pedometers to obtain more objective measures of physical activity, as well as employing actigraphs for more precise assessments of sleep quality. Such methodologies will enable a more scientific and rational elucidation of the relationship between physical activity and sleep quality.**”**

**3. Results:**

***Comment #6. First, it will give more detail of the specific nature of the practical significance of the findings. First, while the significance and nature of the correlations, regression coefficients are well reported, how big these differences are in real world terms (ie, effect sizes) would be an addition.***

**Response:** Thank you for your precious comment. Based on your comments, we supplemented the effect sizes with eta-squared values. The specific changed content is as follows.

**[Pg16, Ln19]- [Pg17, Ln5]: “**Regression analysis indicated that physical activity had a significant effect on sleep quality, with an eta-squared value of 0.293, and that mobile phone addiction similarly affected sleep quality, with an eta-squared value of 0.327. These findings suggest that both physical activity and mobile phone addiction are significant factors influencing sleep quality. Additionally, self-control exerted a significant influence on mobile phone addiction (with an eta-squared value of 0.171) and sleep quality (with an eta-squared value of 0.292), although the effect sizes were slightly smaller compared to physical activity and mobile phone addiction. The study's findings imply that increases in physical activity and self-control may potentially contribute to enhancements in sleep quality and reductions in mobile phone addiction.**”**

***Comment #7.*** ***Add more visual representation: While tables are given, a graphic display to explain the chain mediation model (apart from the flowchart) might be helpful to elucidate the directions of the variables to the audience.***

**Response:** Thank you for your precious comment. Based on your comments, we have added some images that help to clarify the direction of the variables to the audience. The specific changed content is as follows.

**[Pg18, Ln1]- [Pg18, Ln2]: “**The specific paths are presented in Fig 2, Fig 3, Fig 4.**”**

**[Pg18, Ln21]- [Pg18, Ln26]:** “Fig 2 Model of mediating roles of self-control between physical activity and sleep quality; Fig 3 Model of mediating roles of mobile phone addiction between physical activity and sleep quality; Fig 4 Model of chain mediating roles of self-control and mobile phone addiction between physical activity and sleep quality.**”**

**4. Discussion:**

***Comment #8. Address conflicting evidence: Subsequently you cite some papers showing that physical activity has no overall impact on the quality of sleep. Adding on those two aspects would provide a better reason for considering why your study differs, thus enhancing the representation of the work’s credibility.***

**Response:** Thank you for your precious comment. We believe that our study employs a large sample size and encompasses a broad scope of investigations when compared to other studies. Furthermore, we anticipate that our investigation will provide compelling evidence supporting the significant impact of physical activity on sleep quality in younger populations. The specific changed content is as follows.

**[Pg19, Ln11]- [Pg19, Ln14]: “**This investigation utilized a large-scale cross-sectional study to examine the relationship between physical activity and sleep quality within a university student population, contributing to the growing body of evidence suggesting that physical activity exerts a significant influence on sleep quality in young adults.**”**

***Comment #9. Elaborate on cultural context: Because this study concerns Chinese college students, it might have been more useful to pay more attention to potential relaters that could explain sleep, mobile phone addiction, or self-control in some way in this particular group, especially in the contrast with the effect surveyed in Western or other culture.***

**Response:** Thank you for your precious comment. Based on your comments, we have taken into account the cultural differences such as social pressures and cultural expectations faced by Chinese university students and used this to explain their sleep and mobile phone addiction..

**[Pg19, Ln23]- [Pg20, Ln7]: “**Moreover, Chinese college students face unique social pressures and cultural expectations that may affect their sleep patterns and mobile phone habits. The emphasis on academic excellence in Chinese society often results in extended study periods, potentially contributing to sleep deprivation. Additionally, Confucian values, which underscore social harmony, could influence students' self-regulation and perception of mobile phone addiction. Further investigation into these cultural subtleties is essential to understand their implications for the psychological and behavioral health of college students worldwide.**”**

***Comment #10. Explore potential intervention strategies: Nevertheless, such recommendations or ideas for the improvement of sleep quality as an intervention (e.g., decreasing the use of a mobile phone through special university programs) would be more valuable.***

**Response:** Thank you for your precious comment. Based on your comments, we have come up with a number of suggestions and ideas for interventions to improve the quality of sleep among university students, specifically courses on the scientific use of mobile phones and sleep health education, and encouraging university students to participate in sports club activities. The specific changed content is as follows.

**[Pg23, Ln9]- [Pg23, Ln13]: “**Based on the findings of our study, we recommend that universities incorporate courses addressing the scientific use of mobile phones and sleep health education into their curricula. Additionally, we suggest that universities encourage students to engage more actively in sports clubs and extracurricular activities, as these measures may enhance sleep quality among university students.**”**

**5. Limitations and Future Research:**

***Comment #11. Expand the limitations section: If you want to qualify your work you pointed out the cross-sectional design as a weakness, it will better to talk about other variables which could have an influence but were not included in your study for example mental health issues like anxiety or depression.***

**Response:** Thank you for your precious comment. Based on your comments, we believe that we have the following additional limitations to this study that do not take into account other variables that may affect sleep quality, such as mental health issues like anxiety and depression, and environmental factors like lighting and noise. The specific changed content is as follows.

**[Pg23, Ln18]- [Pg23, Ln20]: “**In addition, this study did not account for other variables that might influence sleep quality, such as mental health issues including anxiety and depression, as well as environmental factors like lighting and noise.**”**

***Comment #12. Suggest more detailed future research directions: In discussing future work you say that you still require experimental studies or longitudinal studies etc., it will be more helpful if you were to give some possible set up of such studies like how some reduction in the use of mobile phones can be tested experimentally through randomized controlled trials.***

**Response:** Thank you for your precious comment. Based on your comments, we offer physical interventions such as long-distance running or rock climbing to assess the impact of physical activity on mobile phone addiction and sleep quality. The specific changed content is as follows.

**[Pg24, Ln1]- [Pg24, Ln8]: “**Future research endeavors should incorporate longitudinal studies to elucidate the temporal effects of physical activity on sleep quality. Additionally, the establishment of intervention trials designed to examine the mediating roles of variables such as self-regulatory capacity and cellular phone dependency will provide further insights into the nuanced relationship between physical activity and sleep quality. For instance, intervention trials could be designed to examine the impact of various physical activities, such as long-distance running and rock climbing, on both mobile phone addiction and sleep quality.**”**

***Comment #13. Potential for generalization: Briefly, it is possible to state that the conclusions obtained can be applied to college students in other counties as well as only to the Chinese students depending on the type of the correspondent study.***

**Response:** Thank you for your precious comment. Based on your comments, we have added the relevant note that the conclusions reached in this study, as well as the interventions, can be applied to other countries or only to Chinese university students, depending on the type of the corresponding study. The specific changed content is as follows.

**[Pg23, Ln20]- [Pg24, Ln1]: “**However, the conclusions reached and the proposed intervention strategies in this study may be applicable to university students in other countries, or they may be more specifically relevant to Chinese university students, depending on the nature of the corresponding research.**”**

**Response to Reviewer**

Dear Reviewer:

Thank you for your valuable comments on this study, which provided us with ideas for further analyses of the relationship between physical activity and sleep quality, including post-hoc analyses, bivariate analyses, and so on. Based on the full discussion and understanding of your comments, we spent a great deal of time on revision, and we sincerely hope that the revised manuscript will meet your expectations. Thank you again for your comments and suggestions. In the following, we will respond to your valuable comments point by point.

***Comment #1. Global burden of diseases and sleep prevalence statistics globally or comparative prevalence of China versus US or other countries should be mentioned, instead of US alone. Focused global data or China sleep problems among college students needs to be mentioned.***

**Response:** Thank you for your precious comment. Based on your comments, we have added the appropriate research data report. The specific changed content is as follows.

**[Pg2, Ln14]- [Pg2, Ln20]: “**The overall prevalence of poor sleepers in the Korean general population was 41.0%, with males accounting for 35.6% and females for 46.2% [5].The findings from the German Health Interview and Examination Survey for Adults reveal that approximately one-third of participants reported experiencing significant clinical issues with either initiating or maintaining sleep. Additionally, nearly one-fifth of respondents indicated that they frequently suffer from poor sleep quality [6].**”**

***Comment #2. Physical effects like obesity or mental effects such as anxiety and depression effects due to sleep deprivation missing.***

**Response:** Thank you for your valuable comments. According to your comments, we have added the physical and mental effects of sleep deprivation. The specific changed content is as follows.

**[Pg2, Ln23]- [Pg3, Ln2]: “**Poor sleep quality may lead to chronic diseases such as obesity [9] and cardiovascular disease [10], and it may also trigger depression [11], mood disorders [12], and other health problems.**”**

***Comment #3. More studies should be included where inappropriate or lack of proper guided physical activity can negatively impact sleep or can have complications for sleep, should be considered.***

**Response:** Thank you for your helpful and valuable comments. Based on your comments, we included some research on the effects of lack of physical activity on sleep. The specific changed content is as follows.

**[Pg3, Ln17]- [Pg3, Ln21]: “**The meta-analysis showed an association between lack of physical activity or sedentary behavior and an elevated risk of depression [22], which may also lead to the development of metabolic syndrome [23]. Additionally, prolonged LED screen use may significantly suppress melatonin, which in turn affects the biological clock and leads to sleep problems [24].**”**

***Comment #4. Mentioning author name will be more apt instead of writing some studies.***

**Response:** Thank you for your helpful and valuable comments. Based on your comments, we have quoted the author directly by mentioning her name. The specific changed content is as follows.

**[Pg4, Ln21]- [Pg4, Ln24]: “**The study by Che et al. found that good sleep quality is strongly positively associated with cardiovascular health, metabolic health, immune system health, and cognitive function, including reduced risk of cardiovascular disease, lower incidence of metabolic syndrome[31], etc.**”**

***Comment #5. Is Alzheimer’s disease relevant to the population being studied. More generalized symptoms are mentioned, can be more apt with the population being studied.***

**Response:** Thank you for your helpful and valuable comments. Based on your comments, we have added some research related to college students. The specific changed content is as follows.

**[Pg4, Ln24]- [Pg4, Ln2]: “**College students who engage in physical activity more frequently report lower anxiety levels and greater well-being[32], and those who spend more time on such activities exhibit a higher self-concept.“

***Comment #6. Sleep quality can be further elaborated to sleep latency, timings, duration etc.***

**Response:** Thank you for your constructive and helpful comments. Based on your comments, we have refined the quality of sleep. The specific changed content is as follows.

**[Pg5, Ln2]- [Pg5, Ln6]: “**Unfortunately, poor sleep quality has been a common phenomenon among college students, which manifested by inadequate sleep duration, difficulty falling asleep, daytime dysfunction, and persistent sleepiness. Although poor sleep quality can lead to lower academic performance and mental health issues for these students.**”**

***Comment #7. Sleep quality is mentioned twice.***

**Response:** Thank you for your valuable comments. Based on your comments, we removed duplicates and reorganized the sentences. The specific changed content is as follows.

**[Pg5, Ln13]- [Pg5, Ln15]: “**A recent systematic review study also concluded that regular physical activity can improve sleep quality, reduce sleep latency, and enhance sleep efficiency [36].**”**

***Comment #8 Clarity on mild, moderate or severe physical activity affects sleep quality is needed.***

**Response:** Thank you for your valuable comments. Based on your comments, we are clear about the effect of exercise intensity on sleep quality. The specific changed content is as follows.

**[Pg5, Ln15]- [Pg5, Ln18]: “**Although the relationship between physical intensity and sleep quality lacks experimental evidence, the review demonstrates that moderate physical exercise is beneficial for sleep quality across all age groups within the healthy population [35].**”**

***Comment #9. Mention which kind of exercise or physical activity***

**Response:** Thank you for your valuable comments. Based on your comments, we have consulted the relevant literature and added relevant content.

**[Pg6, Ln1]- [Pg6, Ln3]: “**A strength model demonstrates that self-control, akin to muscle strength, can be enhanced through appropriate exercises, including weightlifting, resistance training, and aerobic activities [27].**”**

***Comment #10. To understand self-control mediating effect, what kind of self-control is referred. For example, Refraining from timings of bed time or other distractions.***

**Response:** Thank you for your valuable comments. Based on your comments, we refined what kind of self-control affects sleep quality. The specific changed content is as follows.

**[Pg6, Ln9]- [Pg6, Ln12]: “**Individuals with diminished self-regulatory capabilities are frequently prone to yielding to immediate temptations in the vicinity of bedtime, which often results in excessive smartphone use and subsequent delays in bedtime. This behavior, in turn, contributes to suboptimal sleep quality [41].**”**

***Comment #11. Factors contributing to self-control apart from physical activity needs to be mentioned. Moreover, type, frequency of physical activity and self-control needs to be addressed.***

**Response:** Thank you for your valuable comments. Based on your comments, we have added research on factors that promote self-control, and types of physical activity. The specific changed content is as follows.

**[Pg6, Ln17]- [Pg6, Ln22]: “**On the one hand, the adoption of a healthy lifestyle, which encompasses regular physical activity, sufficient sleep [42], constructive habits [43], effective emotional regulation [44], and proficient stress management techniques [45], all significantly contribute to the enhancement of an individual's self-control. Regular engagement in chronic physical exercise positively influences executive function, which underpins the capacity for self-regulation [46].**”**

***Comment #12. Specific role of mobile phone addiction on mental and physical health is missing like decrease in melatonin secretion and cognition.***

**Response:** Thank you for your valuable comments. Based on your comments, we have added the specific impact of mobile phone addiction on physical and mental health. The specific changed content is as follows.

**[Pg7, Ln19]- [Pg7, Ln24]: “**Individuals with excessive cell phone usage exhibited significantly reduced daily step counts and reported a higher incidence of neck pain and ocular discomfort. Furthermore, such individuals were more prone to engage in nocturnal cell phone use, which can disrupt circadian rhythms and potentially result in dysregulation of melatonin production, thereby contributing to the onset of emotional disorders, including depression [55].**”**

***Comment #13. Kindly include any study of China to avoid cultural differences or include a study of China and mention global findings***

**Response:** Thank you for your valuable comments. Based on your comments, we have added research related to the Chinese group and a reference to global research. The specific changed content is as follows.

**[Pg8, Ln6]- [Pg8, Ln11]: “**A recent study conducted among Chinese adolescents revealed that the prevalence rates of cell phone addiction and poor sleep quality were 26.2% and 23.1%, respectively. Moreover, the incidence of poor sleep quality was found to be significantly higher within the group exhibiting cell phone addiction [59]. Extensive research has demonstrated that poor sleep quality has emerged as a prominent consequence of excessive cell phone use [60-62].**”**

***Comment #14. Highlight the role of physical activity in reducing mobile dependency***

**Response:** Thank you for your valuable comments. Based on your comments, we have added literature related to the role of physical activity in reducing dependence on mobile phones. The specific changed content is as follows.

**[Pg8, Ln15]- [Pg8, Ln20]: “**Physical exercise has been shown to ameliorate the psychological and mental symptoms associated with cell phone addiction within a study group by modulating the functions of both the endocrine and immune systems [64]. Additionally, engagement in physical activity can enhance the cognitive function of the hippocampus, potentially serving as a mechanism to mitigate mobile phone addiction [65].**”**

***Comment #15. Role of other factor like environment, stress etc. on physical activity and self-control are missing.***

**Response:** Thank you for your valuable comments. Based on your comments, we have added to the literature on the effects of other factors on physical activity and self-control. The specific changed content is as follows.

**[Pg9, Ln8]- [Pg9, Ln12]: “**Social support [68], emotion regulation [44], and stress management [45] all play a role in self-control. Personal factors, including self-motivation [69] and availability of leisure time, alongside environmental factors such as familial support [70] and climatic conditions, are primary determinants affecting an individual's engagement in physical activity.**”**

***Comment #16. Any confounding variable is missing or not highlighted as per limitation of the study needs to be mentioned***

**Response:** Thank you for your valuable comments. Based on your comments, we mentioned the effects of depression, anxiety, and genetics on mobile phone addiction. The specific changed content is as follows.

**[Pg9, Ln15]- [Pg9, Ln17]: “**Depression, anxiety, alterations in brain connectivity, and genetic predispositions may all serve as risk factors for mobile phone addiction [55].**”**

***Comment #17. Lack of evidence of is there for all mediating factors***

**Response:** Thank you for your valuable comments. Based on your comments, we address the relationship between self-control and mobile phone addiction, as well as the effects of physical activity on self-control and mobile phone addiction, and the effects of self-control and mobile phone addiction on sleep quality, and then explore the potential mediating role of self-control and mobile phone addiction in the effects of physical activity on sleep quality. The specific changed content is as follows.

**[Pg9, Ln20]- [Pg10, Ln2]: “**Excessive smartphone use may mediate the relationship between physical inactivity and sleep quality issues among students, suggesting that high levels of smartphone engagement, in conjunction with sedentary behaviors, could exacerbate sleep-related problems [73]. In summary, self-control is highly correlated with cell phone addiction. Moreover, both self-control and cell phone addiction appear to mediate the relationship between physical activity and sleep quality.**”**

***Comment #18. Ethical clearance certificate number, Registration number of the study and Declaration of Helinski missing.***

**Response:** Thank you for your valuable comments. Based on your comments, we refined the ethical review as well as the Declaration of Helinski, and given that our study was a cross-sectional questionnaire study, we did not register the study. The specific changed content is as follows.

**[Pg10, Ln7]- [Pg10, Ln11]: “**The studies involving human participants were reviewed and approved by the Ethics Committee of Chengdu Sport University (〔2023〕150) on October 8, 2023. The study was conducted in accordance with the principles of the Declaration of Helsinki, as revised in 2013.**”**

***Comment #19. Did any method of screening done of the participants or it was only convenient sampling***

**Response:** Thank you for your valuable comments. Based on your comments, and in conjunction with what we've seen previously in our surveys, we've supplemented our screening criteria for participants. The specific changed content is as follows.

**[Pg10, Ln16]- [Pg10, Ln20]: “**The questionnaire included the purpose and method of research, anonymity and confidentiality regarding participation, and the choice to agree or withdraw from participation, participant requirements (1. enrolled students, 2. no physical disabilities, 3. no mental illness), as well as the confirmation that the survey will not be used for purposes other than research.**”**

***Comment #20. Table lacks female percentage***

**Response:** Thank you for your valuable comments. Based on your comments, we have added the percentage of female. The specific changed content is as follows.

**[Pg11, Ln9]:** “Female, n%, 1531（67.3%）.”

***Comment #21.*** ***Was post hoc analysis done?***

**Response:** Thank you for your valuable comments. Based on your comments, we have added the percentage of female. The specific changed content is as follows.

**[Pg13, Ln12] - [Pg13, Ln15]:** “In addition, the study employed chi-square tests (post hoc analyses) to investigate variations in poor sleep quality (PSQI score ≥ 8) and mobile phone addiction detection rates (Sav-Sv score ≥ 32) among university students across different genders and academic years.）.”

**[Pg16, Ln15] - [Pg16, Ln19]:** Post-hoc analyses revealed that female students(χ²=42.68,P<0.001) and those in their fourth year(χ²=23.80,P<0.001)of college were more prone to poor sleep quality, and that the level of mobile phone dependence was statistically higher among female students compared to male students(χ²=14.26,P<0.001).

***Comment #22.*** ***Only unidirectional effect of self-control and mobile phone addiction on sleep is studied , but there could be bidirectional effect also For example, lack of sleep resulting into more usage of mobile phone and lack of self-control.***

**Response:** Thank you for your valuable comments. Based on your comments, we conducted regression analyses to explore bidirectional associations between sleep quality and self-control, mobile phone addiction, and physical activity variables. The specific changed content is as follows.

**[Pg15, Ln16] - [Pg15, Ln21]:** “The stepwise regression analysis was employed to examine the influence of physical activity on sleep quality. Additionally, we assessed the presence of a significant chain mediation effect of self-control and mobile phone addiction in this relationship. Furthermore, we explored the bidirectional associations between sleep quality and the variables of self-control, cell phone addiction, and physical activity using regression analyses.”

**[Pg16, Ln11] - [Pg16, Ln15]:** “There was also a bidirectional effect between sleep quality and self-control, as well as mobile phone addiction. Analyses indicated that sleep quality exhibited a significant negative regression effect on mobile phone addiction (β = –0.541, p < 0.001), and conversely, a significant positive regression effect on mobile phone addiction (β = 0.572, p < 0.001).”

***Comment #23.*** ***Lack of proper discussion on type , intensity, duration and timings which could be best suited for inducing sleep are missing.***

**Response:** Thank you for your valuable comments. Based on your comments, we've added a discussion of the types, intensities, durations, and times that are appropriate for inducing sleep. The specific changed content is as follows.

**[Pg19, Ln14]- [Pg19, Ln21]: “**Majd A. Alnawwar's review indicates that engaging in physical activity during the morning or afternoon may confer greater benefits to sleep quality compared to intense exercise performed at night or in the evening. The study suggests that moderate-intensity aerobic exercises, strength training regimens, as well as mind-body practices like yoga and tai chi, can enhance the quality of sleep. Moreover, the research finds an association between improved sleep quality and a frequency of four to seven weekly sessions of aerobic exercise, coupled with three sessions of moderate-intensity exercise per week.[36]**”**

***Comment #24.*** ***factors affecting self-control are not discussed.***

**Response:** Thank you for your valuable comments. Based on your comments, we have added a discussion of factors that influence self-control. The specific changed content is as follows.

**[Pg21, Ln1]- [Pg21, Ln6]: “**One theoretical framework posits that self-control is a finite internal resource, which, when depleted, impairs one's ability to process information effectively and to regulate cognition, behavior, and emotion. An alternative model suggests that motivation is a primary determinant of self-control failure. Consequently, a variety of factors, including stress, self-control beliefs, motivation, social support, familial influences, and temperament, all contribute to the dynamics of self-control capacity [68]**”**

***Comment #25.*** ***Role of other mediating variables or confounding variable like stress, health, environment, demographics affecting sleep are lacking in discussion. Lifestyle factors，mental disorders，social relations，Physical factors such as pain and fatigue***

**Response:** Thank you for your valuable comments. Based on your comments, we added a discussion of the effects of other mediating or confounding variables on sleep quality. The specific changed content is as follows.

**[Pg22, Ln1]- [Pg22, Ln5]: “**In conjunction with physical activity levels, lifestyle factors such as smoking and dietary habits are recognized as pivotal risk factors affecting sleep quality. Moreover, psychological well-being, including perceived stress and anxiety, exerts a profound influence on the quality of sleep. Additionally, physical factors such as chronic pain and fatigue significantly contribute to the degradation of sleep quality [13].**”**

***Comment #26.*** ***More longitudinal studies or interventional studies are required to prove the chain mediating effect, can be added in future scope.***

**Response:** Thank you for your valuable comments. Based on your comments, we discuss future longitudinal studies as well as intervention trials to further understand the effects of physical activity on sleep quality. The specific changed content is as follows.

**[Pg24, Ln1]- [Pg24, Ln8]: “**Future research endeavors should incorporate longitudinal studies to elucidate the temporal effects of physical activity on sleep quality. Additionally, the establishment of intervention trials designed to examine the mediating roles of variables such as self-regulatory capacity and cellular phone dependency will provide further insights into the nuanced relationship between physical activity and sleep quality. For instance, intervention trials could be designed to examine the impact of various physical activities, such as long-distance running and rock climbing, on both mobile phone addiction and sleep quality.**”**

***Comment #27.*** ***Page numbers are missing***

**Response:** Thank you for your valuable comments. We have critically reviewed all the references you pointed out and cited them on their official websites, adding the appropriate page numbers. The specific changed content is as follows.

**[Pg26, Ln7]- [Pg26, Ln8]:** “Koohsari MJ, Yasunaga A, McCormack GR, Shibata A, Ishii K, Liao Y, et al. Sedentary behaviour and sleep quality. Scientific Reports. 2023;13(1):1180.”

***Comment #28.*** ***Page numbers are missing***

**Response:** Thank you for your valuable comments. We have critically reviewed all the references you pointed out and cited them on their official websites, adding the appropriate page numbers. The specific changed content is as follows.

**[Pg26, Ln40]- [Pg26, Ln42]:** “Li Y, Bai W, Zhu B, Duan R, Yu X, Xu W, et al. Prevalence and correlates of poor sleep quality among college students: a cross-sectional survey. Health and Quality of Life Outcomes.2020;18(1):210.”

***Comment #29.*** ***Page numbers are missing***

**Response:** Thank you for your valuable comments. We have critically reviewed all the references you pointed out and cited them on their official websites, adding the appropriate page numbers. The specific changed content is as follows.

**[Pg26, Ln45]- [Pg27, Ln1]:** “Hu B, Shen W, Wang Y, Wu Q, Li JL, Xu XZ, et al. Prevalence and related factors of sleep quality among Chinese undergraduates in Jiangsu Province: multiple models' analysis. Frontiers in psychology. 2024; 15:1343186.”

***Comment #30.*** ***Vol and page numbers are missing***

**Response:** Thank you for your valuable comments. We have critically reviewed all the references you pointed out and cited them on their official websites, adding the appropriate page numbers. The specific changed content is as follows.

**[Pg27, Ln14]- [Pg27, Ln16]:** “Fukuie T, Inoue K, Yamaguchi A. Lifestyle Elements for Improving Mental and Physical Health in Japanese University Students: Subjective Sleep Quality is a Common Key Factor. American Journal of Lifestyle Medicine. 2023.18(3):303–312.”

***Comment #31.*** ***Page numbers are missing***

**Response:** Thank you for your valuable comments. We have critically reviewed all the references you pointed out and cited them on their official websites, adding the appropriate page numbers. The specific changed content is as follows.

**[Pg27, Ln29]- [Pg27, Ln31]:** “Ding Y, Wan X, Lu G, Huang H, Liang Y, Yu J, et al. The associations between smartphone addiction and self-esteem, self-control, and social support among Chinese adolescents: A meta-analysis. Frontiers in psychology. 2022;13 :1029323.”

***Comment #32.*** ***Page numbers are missing***

**Response:** Thank you for your valuable comments. We have critically reviewed all the references you pointed out and cited them on their official websites, adding the appropriate page numbers. The specific changed content is as follows.

**[Pg28, Ln1]- [Pg28, Ln3]:** “Schmickler JM, Blaschke S, Robbins R, Mess F. Determinants of Sleep Quality: A Cross-Sectional Study in University Students. International journal of environmental research and public health. 2023;20(3):2019.”

***Comment #33.*** ***Page numbers are missing***

**Response:** Thank you for your valuable comments. We have critically reviewed all the references you pointed out and cited them on their official websites, adding the appropriate page numbers. The specific changed content is as follows.

**[Pg28, Ln9]- [Pg28, Ln11]:** “Alnawwar MA, Alraddadi MI, Algethmi RA, Salem GA, Salem MA, Alharbi AA. The Effect of Physical Activity on Sleep Quality and Sleep Disorder: A Systematic Review. Cureus Journal of Medical Science. 2023;15(8): e43595.”

***Comment #34.*** ***Page numbers are missing***

**Response:** Thank you for your valuable comments. We have critically reviewed all the references you pointed out and cited them on their official websites, adding the appropriate page numbers. The specific changed content is as follows.

**[Pg28, Ln21]- [Pg28, Ln23]:** “Ye J, Jia X, Zhang J, Guo K. Effect of physical exercise on sleep quality of college students: Chain intermediary effect of mindfulness and ruminative thinking. Frontiers in psychology.2022;13:987537.”

***Comment #35.*** ***Page numbers are missing***

**Response:** Thank you for your valuable comments. We have critically reviewed all the references you pointed out and cited them on their official websites, adding the appropriate page numbers. The specific changed content is as follows.

**[Pg31, Ln7]- [Pg31, Ln9]:** “Li Y, Guo K. Research on the relationship between physical activity, sleep quality, psychological resilience, and social adaptation among Chinese college students: A cross-sectional study. Frontiers in psychology. 2023;14 :1104897.”

***Comment #36.*** ***Page numbers are missing***

**Response:** Thank you for your valuable comments. We have critically reviewed all the references you pointed out and cited them on their official websites, adding the appropriate page numbers. The specific changed content is as follows.

**[Pg31, Ln36]- [Pg31, Ln38]:** “Gao C, Chapagain NY, Scullin MK. Sleep Duration and Sleep Quality in Caregivers of Patients With Dementia A Systematic Review and Meta-analysis. Jama Network Open. 2019;2(8): e199891.”

***Comment #37.*** ***Page numbers are missing***

**Response:** Thank you for your valuable comments. We have critically reviewed all the references you pointed out and cited them on their official websites, adding the appropriate page numbers. The specific changed content is as follows.

**[Pg32, Ln2]- [Pg32, Ln5]:** “Rodriguez-Larrad A, Manas A, Labayen I, Gonzalez-Gross M, Espin A, Aznar S, et al. Impact of COVID-19 Confinement on Physical Activity and Sedentary Behaviour in Spanish University Students: Role of Gender. International journal of environmental research and public health.2021;18(2):369.”

***Comment #38.*** ***Page numbers are missing***

**Response:** Thank you for your valuable comments. We have critically reviewed all the references you pointed out and cited them on their official websites, adding the appropriate page numbers. The specific changed content is as follows.

**[Pg32, Ln9]- [Pg32, Ln11]:** “Xiang M-Q, Lin L, Wang Z-R, Li J, Xu Z, Hu M. Sedentary Behavior and Problematic Smartphone Use in Chinese Adolescents: The Moderating Role of Self-Control. Frontiers in psychology. 2020; 10:3032.”

***Comment #39.*** ***Page numbers are missing***

**Response:** Thank you for your valuable comments. We have critically reviewed all the references you pointed out and cited them on their official websites, adding the appropriate page numbers. The specific changed content is as follows.

**[Pg32, Ln15]- [Pg32, Ln17]:** “Zhao Z, Kou Y. Effect of short video addiction on the sleep quality of college students: chain intermediary effects of physical activity and procrastination behavior. Frontiers in psychology.2024;14:1287735.”
